# ﻿Erica L. (Ericaceae): homonyms amongst published names for African species and proposed replacement names

**DOI:** 10.3897/phytokeys.236.110498

**Published:** 2023-12-20

**Authors:** E. Charles Nelson, E. G. H. Oliver, Michael D. Pirie

**Affiliations:** 1 Tippitiwitchet Cottage, 255A Broadgate, Sutton St Edmund PE12 0LT, UK Unaffiliated Spalding United Kingdom; 2 Department of Botany and Zoology, University of Stellenbosch, Private Bag X1, Stellenbosch, 7602 Matieland, South Africa University of Stellenbosch Stellenbosch South Africa; 3 University Museum, University of Bergen, Postboks 7800, NO-5020 Bergen, Norway University of Bergen Bergen Norway

**Keywords:** Cape heaths, *Erica*, Hans Dulfer, International register of heather names, nineteenth-century English nursery catalogues, nomenclature

## Abstract

In support of ongoing taxonomic work on the large and complex flowering plant genus *Erica* (Ericaceae), we document nineteen pairs of homonyms representing currently used illegitimate names. We provide replacements for thirteen names and new typifications for five. We relegate five names to synonymy: *Ericaaemula* Guthrie & Bolus under *Ericadistorta* Bartl.; *Ericaarmata* Klotzsch ex Benth. under *Ericaumbrosa* H. A. Baker; *Ericacapensis* T.M. Salter under *Ericaturbiniflora* Salisb.; *Ericalanata* Andrews under *Ericaflaccida* Link; and *Ericatomentosa* Salisb. under *Ericavelutina* Bartl. Finally, we suggest conservation of *Ericaaristata* Andrews. The new names are: *Ericaadelopetala* E.C. Nelson & E.G.H. Oliv. replacing *Ericainsignis* E.G.H. Oliv.; *Ericabombycina* E.C. Nelson & Pirie replacing *Ericaniveniana* E.G.H. Oliv.; *Ericaconcordia* E.C. Nelson & E.G.H. Oliv. replacing *Ericaconstantia* Nois. ex Benth.; *Ericadidymocarpa* E.C. Nelson & E.G.H. Oliv. replacing *Ericarugata* E.G.H. Oliv.; *Ericagalantha* E.C. Nelson & E.G.H. Oliv. replacing *Ericaperlata* Benth.; *Ericamallotocalyx* E.C. Nelson & E.G.H. Oliv. replacing *Ericaflocciflora* Benth.; *Ericanotoporina* E.C. Nelson & E.G.H. Oliv. replacing *E.autumnalis* L.Bolus; *Ericaoliveranthus* E.C. Nelson & Pirie replacing *Ericatenuis* Salisb.; *Ericaoraria* E.C. Nelson & E.G.H. Oliv. replacing *Ericaspectabilis* Klotzsch ex Benth.; *Ericaoresbia* E.C. Nelson & E.G.H. Oliv. replacing *Ericademissa* Klotzsch ex Benth.; *Ericapoculiflora* E.C. Nelson & E.G.H. Oliv. replacing *Ericastenantha* Klotzsch ex Benth.; *Ericarhodella* E.C. Nelson & E.G.H. Oliv. replacing *Ericarhodantha* Guthrie & Bolus; *Ericasupranubia* E.C. Nelson & Pirie replacing *Ericapraecox* Klotzsch.

## ﻿Introduction

The nomenclatural history of the genus *Erica* L. *sensu lato* (Oliver 2000, 2012) is complicated by the extraordinary fashion in western Europe for cultivating “Cape heaths”, the English name generally given to plants derived from the *Erica* species endemic to southern Africa. *Erica*-mania commenced in the last decade of the 18^th^ century, burgeoned in the early 1800s and petered out in the middle of that century (Nelson and Pirie 2022). A consequence of the horticultural interest was a proliferation of names, applied often in a haphazard manner by nurserymen and gardeners, as well as by botanists. Many names were first published in nurserymen’s and gardeners’ catalogues and in horticultural periodicals, often with accompanying descriptions or diagnoses (Nelson and Oliver 2004; Nelson and Small 2004–2005; see also Reveal (2012)).

Work carried out between 1995 and 2004 by ECN for The Heather Society (established in 1963, formally dissolved in 2020) on the second volume of the International Register of Heather Names (Nelson and Small 2004–2005) necessarily included an extensive survey of nineteenth-century horticultural publications, resulting in the accumulation of more than 6,000 names, at all ranks and many times that number of bibliographic citations, for *Erica* taxa of African origin. This information was collated in a database, complementing a similar one for the so-called “hardy heaths” – *Andromeda* L., *Calluna* Salisb., *Daboecia* D. Don and species of *Erica* endemic in Europe, Macaronesia and western Asia (Nelson and Small 2000). We are currently working to make this resource openly accessible, particularly through integration with the World Flora Online (WFO 2023; Elliot et al. in prep.). The database provides an invaluable bibliographic tool, much more comprehensive and, for a genus of more than 800 species, more practical and detailed than any existing botanico-bibliographic indexes including such standard publications as “Index Kewensis” which only provided publication information for protologues. Inevitably a database of such a comprehensive nature revealed a scattering of hitherto unsuspected, or inadvertently overlooked, problems with the established and currently accepted names of African *Erica* species.

Some of these problems do not arise, in fact, from the unearthing of long-buried names (most of the binomials discussed here were recorded by Dulfer (1965)), but rather from a better understanding of the history of certain publications and more accurate information about dates of publication of, for example, George Bentham’s (1839) treatment of *Erica* and related genera (Nelson 2005) and Henry Cranke Andrews’s multi-volume works, Coloured Engravings of Heaths (Cleevely and Oliver 2002) and The Heathery (Cleevely et al. 2003).

Although changes in current names are rarely welcomed, particularly outside the taxonomic community, by following the rules set out in the International Code of Nomenclature for Algae, Fungi and Plants (Shenzhen Code) 2018 (Turland et al. 2018) (hereafter ICN (Shenzhen Code) 2018), we can reflect the state of knowledge in the field and maintain overall stability in nomenclature. In this paper, we provide replacement names for homonyms and clarify typifications where necessary.

## ﻿Homonyms in *Erica*

The Heather Society’s database revealed pairs of homonyms within *Erica* where the one in current use (compare Oliver and Oliver (2003); Oliver (2012)) is not the earliest published. The need to replace these later names was signalled in the four published parts of the International Register of Heather Names, volume 2, African Species, Hybrids and Cultivars (Nelson and Small 2004–2005). These names are listed (as summarised in Table 1) and discussed and, where necessary, replacements are provided when an alternative validly published name is not available.

**Table 1.** Homonym pairs in *Erica* in alphabetic order with dates of publication.

Specific epithet	Author(s) and date of currently accepted binomial	Author(s) and date(s) of earlier binomial
*aemula*	Guthrie and Bolus (1905)	Rollisson (1855)
*aristata*	Andrews (1807)	Salisbury (1796)
*armata*	Klotzsch ex Bentham (1839)	Sprengel (1825)
*autumnalis*	L. Bolus (1923)	Hort. ex Bentham (1839)
*capensis*	Salter (1935)	Regel (1842)
*constantia*	Noisette ex Bentham (1839)	Hort. ex Sinclair (1825)
*demissa*	Klotzsch ex Bentham (1839)	Hort. ex Sinclair (1825)
*flocciflora*	Bentham (December 1839)	Tausch (October 1839)
*insignis*	Oliver (1981)	Hort. (1853)
*lanata*	Andrews (1806)	Wendland (1798)
*niveniana*	Oliver (2000)	Hort. ex Loudon (1830)
*perlata*	Bentham (1839)	Sinclair (1825)
*praecox*	Klotzsch (1838)	Hort. ex Sinclair (1825)
*rhodantha*	Guthrie and Bolus (1905)	Regel (1842)
*rugata*	Oliver (2000)	Hort. ex Sinclair (1825)
*spectabilis*	Klotzsch ex Bentham (1839)	Waitz (1805)
*stenantha*	Klotzsch ex Bentham (1839)	Sweet (1830)
*tenuis*	Salisbury (1802)	Moench (1802)
*tomentosa*	Salisbury (1802)	Masson (1776)

It should be noted that nurserymen’s catalogues during the 19^th^ and 20^th^ centuries were usually annual publications, reissued with minor amendments, deletions and additions, year after year, decade after decade. The dated catalogues noted in this paper are examples only – they must not be assumed to be the first, the last or the only issue containing a particular name.

### ﻿1. *Ericaaemula* Rollisson (1855), non Guthrie and Bolus (1905)

This binomial first appeared in print within an advertisement inserted by Messrs William Rollisson & Sons of Tooting, London, in “The Gardeners’ Chronicle”: 218 (7 April 1855). A brief description, noting the “fine bright crimson” flowers, was included that validates the name. Subsequently, it was published in several other English nurserymen’s catalogues (see Nelson and Small (2004: pt 1: 11)): for example, in William Rollisson and Sons’ catalogue for 1877, which company claimed it as a hybrid raised and sent out by them; James Fraser, Lea Bridge Road Nursery, for 1866–1867; E. G. Henderson and Son for Autumn 1871; James Veitch and Sons for 1873–1874; and B. S. Williams for 1881. Compilers of gardening dictionaries soon included this heath in their works including Hereman (1868: 217) and Wright (ca. 1907: 325).

Guthrie and Bolus (1905), presumably unaware of the previous publication of the epithet *aemula* within *Erica*, used the same epithet for a white-blossomed species that inhabits rocky, coastal slopes and inland, marshy, high mountain plateaux in the Western Cape, from Bainskloof to the Steenbras Mountains (Oliver and Oliver 2000: 429). The species was illustrated by Schumann et al. (1992: 104) and is listed amongst the recognised plant species of southern Africa by Oliver and Oliver (2003). However, it was subsequently treated as a local variant from Gordon’s Bay of *E.distorta* Bartl. (Oliver and Forshaw 2012). We follow the latter taxonomic opinion and treat *E.aemula* as a heterotypic synonym of *E.distorta* without providing a replacement name.

#### ﻿ Erica distorta

Taxon classificationPlantaeEricalesEricaceae

Bartl., Linnaea 7: 644. 1832

C77C3553-0762-5B78-9FEA-CAF0E9DA26DE

Erica aemula Guthrie & Bolus, Fl. Capensis 4,1: 118. 1905 [wfo-0000671313], nom. illeg., non Ericaaemula Rollisson, Gard. Chron. 1855: 218 (1855) [wfo-1200040659]. Type. South Africa. “Bains Kloof“ [Bainskloof], *Cummings 171* BOL [BOL137142 [https://plants.jstor.org/stable/10.5555/al.ap.specimen.bol137142], syntype; “Fish Hoek, Gordon’s Bay“, *Guthrie 3108* BOL (BOL137141) [https://plants.jstor.org/stable/10.5555/al.ap.specimen.bol137141], syntype.

##### Type.

South Africa, “Auf Felsenrücken in der Kluft nach der Platte des Tafelberges in vierter Höhe” (lectotype †GOET destroyed, *fide* annotation by N. E. Brown on K000314148; isolectotype [fragments only], “ad Cap. b. Spei in monte tabulari leg. Ecklon. Hb. Bg. 1841” K [K000314148].

### ﻿2. *Ericaaristata* Salisb. (1796) [wfo-1000053511], non Andrews (1807) [wfo-0000671412]

Richard Anthony Salisbury’s (1761–1829) catalogue (Salisbury 1796) of the plant collection in his own garden at Chapel Allerton, outside Leeds in Yorkshire, England, gave the name *Ericaaristata* for a plant from the Cape of Good Hope, introduced by the nurseryman James Lee (1715–1795). The accompanying diagnosis validates the binomial, but is quite inadequate to identify the species, which evidently had not bloomed (there is no description of the flowers, only the ternate, linear leaves). Guthrie and Bolus (1905) and Dulfer (1965) placed Salisbury’s name in synonymy under *E.banksii* Andrews. No herbarium specimens, determined by Salisbury, of his *E.aristata* are known so that equation is extremely dubious, especially given the brevity of the protologue.

A decade later the same binomial was employed by Henry Cranke Andrews (fl. 1794–1830) when he illustrated and described the plant that currently bears this name, an inhabitant of the Kleinrivier Mountains (Andrews 1807: t. 152; 1809a: t. 147). It is an erect, semi-spreading shrublet, to 0.5 m tall, producing large (to 25 mm long), tubular-inflated flowers that are very sticky and have spreading lobes; the corolla is longitudinally striped dark and light pink (Oliver and Oliver 2000).

This species was illustrated by Schumann et al. (1992: 80) and is listed amongst the currently recognised plant species of southern Africa (Oliver and Oliver 2003; Oliver 2012: 489). This species is a very well-known plant due to its striking floral morphology. It is the first *Erica* species for which pollination by long-proboscid flies has been demonstrated (Lombardi et al. 2021). Changing its name would cause considerable confusion, so a proposal will be submitted for the conservation of *E.aristata* Andrews over *E.aristata* Salisb.

### ﻿3. *Ericaarmata* Spreng. (1825), non Benth. (1839)

*Ericaarmata* was validly published by Sprengel (1825: 2: 184), but Guthrie and Bolus (1905: 86) and Dulfer (1965: 53) placed it in synonymy under *E.sparrmanii* L.f., despite upholding *E.armata* Klotzsch ex Benth. (1839) as a distinct species. Thus, *Ericaarmata* Klotzsch ex Benth. (1839: 672) is an illegitimate later homonym. The species to which the binomial is currently applied (see Oliver and Oliver (2003: 427); Schumann et al. (1992: 100); Oliver (2012: 489)) is an erect shrublet, to 0.5 m tall, bearing broadly urn-shaped to tubular, hairy, pink flowers with exserted anthers; it occurs on rocky slopes. Fortunately, a later synonym for it is available, *Ericaumbrosa* (Baker 1961).

#### ﻿ Erica umbrosa

Taxon classificationPlantaeEricalesEricaceae

H.A.Baker, J. S. Afr. Bot. 27: 267. 1961

893F8E5B-5124-5196-A716-43CBB866D0C4

E. armata Klotzsch ex Benth., Prodr. [A. P. de Candolle] 7(2): 672. 1839 [wfo-0000671419], nom. illeg., non Spreng., Syst. Veg. 2: 184. 1825 [wfo-0000671418]; Guthrie and Bolus, Fl. Capensis 4,1: 113. 1905; Dulfer, Ann. Naturhist. Mus, Wein 68: 67. 1965; Oliver, Strelitzia 29: 489. 2012. Type. South Africa. “in herb. reg. Berol. … in montibus prov. Worcester et Stellenbosch”, Masson, Niven, Drège (lectotypes B destroyed; isolectotype K [*Niven 158*] (det. E.G.H. Oliver) K000314208).

##### Note.

Specimens in other herbaria collected by Masson and Drège are variously labelled as syntypes or isosyntypes, but their identity has not been confirmed: GDC (Masson, F., s.n., G00494351 https://plants.jstor.org/stable/10.5555/al.ap.specimen.g00494351); GDC (Drège, J.F., s.n., G00494352 https://plants.jstor.org/stable/10.5555/al.ap.specimen.g00494352); HBG (Drège, J.F., s.n., HBG515307 https://plants.jstor.org/stable/10.5555/al.ap.specimen.hbg515307); TUB (Drège, J.F., s.n., TUB003182 left-hand specimen only https://plants.jstor.org/stable/10.5555/al.ap.specimen.tub003182); S (Drège, J.F., s.n., S08-5237 https://plants.jstor.org/stable/10.5555/al.ap.specimen.s08-5237).

##### Type.

South Africa. “Caledon, Elandskloof, Villiersdorp, on a steep S-facing slope in shade in kloof with a large waterfall, 3 April 1961, *E.G.H. Oliver 1423* (holotype: BOL; isotype: NBG-0199392-1 [https://plants.jstor.org/stable/10.5555/al.ap.specimen.nbg0199392-1].

### ﻿4. *Ericaautumnalis* Hort. ex Benth. (1839), non L.Bolus (1923)

This binomial was published under the entry for *Ericaformosa* Thunb. with a validating diagnostic phrase by Bentham (1839) who attributed the name to horticulturists. Bentham appended an asterisk indicating that he deemed it to be a horticultural hybrid. Regel (1842) attributed the same name to English gardeners. It was not included in “Index Kewensis”, nor was it taken up or listed by Guthrie and Bolus (1905). On the other hand, Dulfer (1965) included the name, for a hybrid, following Regel.

Bolus (1923) probably overlooked Bentham’s use of the binomial and published it for a Western Cape species found on moist slopes at middle altitude distributed from the Hottentots Holland Mountains to Kogelberg (Dulfer 1964: 146; 1965: 92; Oliver and Oliver 2000: 430; Sieben et al. 2004; Oliver 2012: 490). This species was illustrated by Schumann et al. (1992: 146) and is listed amongst the currently recognised plant species of southern Africa (Oliver and Oliver 2003: 427; Oliver 2012: 490).

A new name is required for the taxon and the new epithet alludes to the autumn, which is the species’ main, Southern-Hemisphere flowering season.

#### ﻿ Erica notoporina

Taxon classificationPlantaeEricalesEricaceae

E.C.Nelson & E.G.H.Oliv. nom. nov.

D1B673B1-36C1-5365-8D50-9F725E6E47F0

urn:lsid:ipni.org:names: 77327519-1

pro Ericaautumnalis L.Bolus, Ann. Bolus Herb. 3: 178. 1923 [wfo-0000671451], nom illeg., non E.×autumnalisHort. ex Benth., Prodr. [A. P. de Candolle] 7(2): 659 (1839) [wfo-1000053512]; Regel, Verh. Vereins Beförd. Gartenbaues Königl. Preuss. Staaten 16: 307. 1842; Regel, Kult. Aufz. Eriken, 147. 1843; Dulfer, Ann. Naturhist. Mus, Wein 68: 151 (1965).

##### Type.

South Africa, “Bought in Adderly St., Cape Town”, 2 February 1922, *N.S. Pillans 16784*: (holotype: BOL [BOL-137249 (https://plants.jstor.org/stable/10.5555/al.ap.specimen.bol137249); isotype BOL [BOL-137250]).

### ﻿5. *Ericacapensis*Regel (1842), non T.M.Salter (1935)

In his monograph on *Erica*, Eduard August von Regel (1815–1892) described a plant named *E.capensis* (Regel 1842: 318, 1843: 158) and the binomial has had a sporadic existence since the mid-19^th^ century. Guthrie and Bolus (1905) did not list Regel’s use of the epithet *capensis*. The binomial reappeared in the early 20^th^ century in, for example, the seed-list of the French nursery Vilmorin Andrieux & Cie for 1922–1923 and was recorded by Dulfer (1965: 151) who opined that Regel’s name was synonymous with *E.pelviformis* Salisb. (= *E.mauritanica* L.).

Salter (1935) employed the same binomial for a species found in marshes at low altitude on the Cape Peninsula (Dulfer 1964: 145, 146; 1965: 90; Oliver and Oliver 2000; Oliver 2012: 493). This species was illustrated by Schumann et al. (1992: 144), and is listed amongst the currently recognised plant species of southern Africa by Oliver and Oliver (2003: 429).

Oliver and Oliver (2003: 429, 447) placed Salter’s name in synonymy under *Ericaturbiniflora* Salisb. (wfo-0000673478). As that name was validly published and pre-dates Salter’s by more than a century, it is the correct name for the taxon.

#### ﻿ Erica turbiniflora

Taxon classificationPlantaeEricalesEricaceae

Salisb., Trans Linn. Soc. 6: 377. 1802

D310F9E3-6EFF-5F50-9C11-DCCDE3A90361

Erica capensis T.M.Salter, J. S. Afr. Bot. 1: 34. 1935 [wfo-0000671621], nom. Illeg., non Regel, Verh. Vereins Beförd. Gartenbaues Königl. Preuss. Staaten 16: 318 (1842) [wfo-1000053513], 158. 1843. Type: South Africa. “Cape Peninsula, marshes on lower Hout and Klaasjager River”, 14 February 1934, *T.M. Salter 4292* (holotype: BOL-137252 [https://plants.jstor.org/stable/10.5555/al.ap.specimen.bol137252]).

##### Type.

Without locality, *Hibbert ex herb. Salisbury* (lectotype, designated here: K [K-314663]).

### ﻿6. *Ericaconstantia* Hort. Ex G.Sinclair (1825), non Nois. Ex Benth. (1839)

A Cape heath named “Erica Constantia” was included by Messrs Lee & Kennedy in a manuscript list of species that had been introduced into cultivation by the firm up to 1808 (see Nelson and Oliver (2004: 138)). The binomial has been traced in print in Conrad Loddiges & Sons’ 1818 catalogue and, three years later, in Johann Heinrich Friedrich Link’s (1767–1851) list of plants in Berlin Botanic Garden (Link 1821: 374). Neither of those publications contained a description, but George Sinclair’s (1787–1834) catalogue of the heaths (Sinclair 1825) grown in the Duke of Bedford’s garden at Woburn, Bedfordshire, England, did include a description (based on at least one living plant, although Sinclair had not observed this in bloom and a herbarium specimen). Sinclair (1825) attributed the name to “Hortulanis” (gardeners) and described the heath as having leaves in threes, with bell-shaped flowers in terminal inflorescences, with awnless anthers. He did not provide (as he usually did) the colour of the corolla because he had only seen a dried specimen. The name is also known from at least eight other 19^th^-century works, including those of Bentham (1839: 666), Regel (1842: 300) who remarked “Die als E.constantia in deutschen Gärten gehende Pflanze gehört zur E. trivialis” and Hereman (1868: 218) whose description indicated that the cultivated plant bore purple flowers.

However, Bentham (1839: 672) also described a species, stated to have been named *Ericaconstantia* by Noisette – presumably the French horticulturist Jean Claude Noisette (1772–1849) – on the basis of one of Klotzsch’s specimens, then in the Berlin Herbarium and destroyed during the Second World War. Guthrie and Bolus (1905: 115) accepted this name, having seen the type material in Berlin “ex horto [Lee &] Kennedy, 1816”, but overlooked the earlier usage, whereas Dulfer (1964: 108, 137; 1965: 67) chose to ignore the other use of the same binomial (including as a synonym for his E.simulansvar.trivialis (Klotsch ex Benth.) Dulfer [wfo-0000673262]) and accepted Bentham’s second application of the name, citing a specimen collected by Esterhuysen (*28188*) from “Oudensberg” [sic. Audebsberg], Worcester District, Western Cape, as matching Bentham’s protologue.

*Ericaconstantia* Nois. ex Benth. is currently applied to a white-flowered heath from rocky slopes at high altitude, ranging from the Hex River Mountains to Klein Swartberg (Oliver and Oliver 2000, 2003: 430; Oliver 2012: 492). It is clearly not the purple-flowered taxon cultivated in European gardens during the 19^th^ century.

Here, we designate the Esterhuysen collection cited by Dulfer (1965) as neotype to fix the application of the name in its current sense and provide a replacement name for the species. One meaning of the Latin word *constantia* is harmony, although whether that was also the intended meaning of Lee & Kennedy’s epithet cannot be determined. *Concordia* also means harmony.

#### ﻿ Erica concordia

Taxon classificationPlantaeEricalesEricaceae

E.C.Nelson & E.G.H.Oliv. nom. nov.

672E7273-8B4D-5952-AA97-594BE4C1DF74

urn:lsid:ipni.org:names: 77327521-1

pro E.constantia Nois. ex Benth., Prodr. [A. P. de Candolle] 7(2): 672. 1839 [wfo-0000671735], nom. illeg., non Hort. ex G.Sinclair, Hort. eric. woburn.: 6, 32. 1825 [wfo-1000055091]; Guthrie and Bolus, Fl. Capensis 4,1: 115–116. 1905; Dulfer, Ann. Naturhist. Mus, Wein 68: 67–68. 1965.

##### Type.

Without locality or collector, *Herb. Klotzsch* (holotype: B, destroyed). South Africa, Audensberg, S slopes. 15 February 1959, *E.E. Esterhuysen 28188* (neotype, designated here, NBG [NBG-0265661-0]).

### ﻿7. *Ericademissa* Hort. ex G.Sinclair (1825), non Klotzsch ex Benth. (1839)

“Dwarf green-flowered heath” was the English name used by Sinclair (1825) when he described a plant cultivated in England in the early 19^th^ century under the name *Ericademissa*. The name (as “*demisa*”) was included in Messrs Lee and Kennedy’s manuscript list, mentioned above (see Nelson and Oliver (2004: 138)), of Cape heaths that had been introduced into cultivation by the firm up to 1808. Within a year of this, the name was printed by Donn (1809), Cushing (1814: 210) and in Conrad Loddiges and Sons’ catalogue for 1818.

Sinclair’s description (1825) is more than adequate to validate the name, which Dulfer (1965: 29) noted, perhaps correctly, as a synonym of *Ericacoccinea* L., no doubt following Sinclair’s grouping of the species (Sinclair 1825: 35). There is a specimen so named in LIV, but we have not examined it.

Meanwhile, Bentham (1839) chose to adopt a name proposed by Klotzsch and, thus, published the same binomial for an entirely different species with white to rosy-pink flowers. This is distributed on the lower to middle slopes of the Swartberg as far east as Grahamstown (Oliver and Oliver 2000). This species was illustrated by Schumann et al. (1992: 137) and is listed amongst the currently recognised plant species of southern Africa (Oliver and Oliver 2003: 432), but clearly is not the “dwarf, green-flowered” heath known to Sinclair.

A new epithet, derived from the Greek compound ορεσβιος (*oresbios*) meaning living on mountains (Stearn 1973), is published here and alludes to the species’ habitat in “rocky veld on the Swartberg” (Schumann et al. 1992: 137).

#### ﻿ Erica oresbia

Taxon classificationPlantaeEricalesEricaceae

E.C.Nelson & E.G.H.Oliv. nom. nov.

AC1C60D6-8A56-56FB-85B2-D934D191A1AD

urn:lsid:ipni.org:names: 77327522-1

pro E.demissa Klotzsch ex Benth., Prodr. [A. P. de Candolle] 7(2): 666. 1839 [wfo-0000671862], nom illeg., non Hort. ex G. Sinclair Hort. eric. woburn.: 8. 1825 [wfo-0000671861]; Benth., Prodr. [A. P. de Candolle] 7(2): 621. 1839; Guthrie and Bolus, Fl. Capensis 4,1: 47. 1905; Dulfer, Ann. Naturhist. Mus, Wein 68: 86–87. 1965.

##### Type.

South Africa. “Uitenhaag [Uitenhage], in monitbus Vanstadensrivier”, 1000–4000[ft], *C.F. Ecklon and C.L.P. Zeyher s.n.* (syntype: not traced); South Africa, “flum. Camtoo [Gamtoos]”, *Masson s.n.* (syntype: not traced), *Burchell 4709* (syntype: HAL [https://plants.jstor.org/stable/10.5555/al.ap.specimen.hal0135770]).

### ﻿8. *Ericaflocciflora*Tausch (1839), non Benth. (1839)

This is an instance of two names published within weeks of each other. Tausch’s binomial was published on 28 October 1839, more than a month before Bentham’s and, thus, has priority. Unfortunately, Dulfer (1965) consistently misquoted (as 1838) the publication date of Bentham’s treatment of *Erica* published in the second part of volume 7 of Augustin Pyramus de Candolle’s *Prodromus*. Late December 1839 is accepted as being the correct publication date for volume 7 part 2, although standard sources (e.g. Stafleu and Cowan (1976)) contain contradictory dates (for discussion, see Nelson (2005)). The first part of volume 7 was issued in 1838, but it does not contain the Ericeae.

Given the fact he had an incorrect (earlier) date of publication, Dulfer (1965: 98, 140) maintained Bentham’s binomial as the correct name for the taxon and disregarded Tausch’s name (which he stated was synonymous with *Ericadaphniflora* Salisb.).

*Ericaflocciflora*, as described by Bentham, occurs on the dry, lower slopes and rocky foothills of the Kouga Mountains and has a cream corolla with a distinctive woolly calyx (Oliver and Oliver 2000). This species was illustrated by Schumann et al. (1992: 157) and is listed amongst the currently recognised plant species of southern Africa (Oliver and Oliver 2003: 434).

Irrespective of synonymy, Bentham’s binomial is illegitimate because it is a later homonym. The new epithet that we provide below continues the allusion to *floccus* (Latin: tuft of woolly hairs) by adopting a Greek equivalent, μαλλωτoς (*mallotos*), fleecy.

#### ﻿ Erica mallotocalyx

Taxon classificationPlantaeEricalesEricaceae

E.C.Nelson & E.G.H.Oliv. nom. nov.

966A3BC7-BD92-5B29-9FE5-99178953EE6C

urn:lsid:ipni.org:names: 77327523-1

pro E.flocciflora Benth., Prodr. [A. P. de Candolle] 7(2): 660. 1839 (late December) [wfo-0000672068], nom. illeg., non Tausch, Flora Bot. Zeit. Regensb. 22: 629. 1839 (28 October) [wfo-0000672067]; Guthrie and Bolus, Fl. Capensis 4,1: 310 (1905); Dulfer, Ann. Naturhist. Mus, Wein 68: 98 (1965).

##### Type.

South Africa. “In colonia capensi” (cit. Bentham 1839) [‘on a rocky hill near Groot River, Uniondale Div.’], 14 March 1814, *W.J. Burchell 4992* (lectotype, here designated, K (K-314571 [https://plants.jstor.org/stable/10.5555/al.ap.specimen.k000314571]; isolectotype, G)

### ﻿9. *Ericainsignis* Hort. (1853), non E.G.H. Oliv. (1981)

This binomial, overlooked by the compilers of “Index Kewensis”, has been traced in no fewer than six publications issued during the latter half of the 19^th^ century including the “Journal of the Royal Horticultural Society” (1853: **8**: xl) and “Revue horticole” (1882: **54**: 219–220). Accompanying descriptions indicated it was applied to a scarlet-blossomed heath. It was also traced in catalogues issued by the following British and New Zealand nurseries: James Fraser, Lea Bridge Road Nursery for 1866–1867; William Rollisson & Sons for 1877; B. S. Williams for 1881; James Dickson & Sons, Newton Nurseries, for 1884; Nairn & Sons, Christchurch (New Zealand) for 1896. It is highly improbable that the plant cultivated at least until the end of the 19^th^ century in European and New Zealand gardens was the same species as that first collected by Stokoe in 1935 and described by Oliver (1981). It inhabits rock crevices on upper, north-facing slopes of mountains including the Anysberg and Groot Swartberg and has remarkable flowers with a very small corolla (± 5 mm) concealed within a greatly extended calyx (± 20 mm). It was illustrated by Schumann et al. (1992: 215) and is listed amongst the currently recognised plant species of southern Africa (Oliver and Oliver 2003: 436).

The new epithet alludes to the concealed corolla (from Greek: αδελoς (adelos = unseen), πεταλov (petalon = leaf, i.e. petal)) and echoes the name of the section to which this species and *E.nabea* Guthrie and Bolus were assigned.

#### ﻿ Erica adelopetala

Taxon classificationPlantaeEricalesEricaceae

E.C.Nelson & E.G.H.Oliv. nom. nov.

67A6AB38-2903-526F-8C6C-ED0212E96494

urn:lsid:ipni.org:names: 77327524-1

pro E.insignis E.G.H. Oliv., Bothalia 13: 446. 1981 [wfo-0000672329], nom. illeg., non hort. in J. Roy. Hort. Soc. 8: xl. 1853 [wfo-1000053515]; Rev. hort. 54: 219–220. 1882.

##### Type.

South Africa, “Swartberg, north slopes below Kangoberg”, 1,400 m, 11 December 1979, *E.G.H. Oliver 7469* (holotype, NBG [as STE] [https://plants.jstor.org/stable/10.5555/al.ap.specimen.nbg0133788-0]; isotypes K, PRE).

### ﻿10. *Ericalanata* J.C. Wendl. (1798), non Andrews (1806)

Messrs Lee & Kennedy had employed the name “Ericalanata” in the manuscript list, mentioned previously (see Nelson and Oliver (2004: 138)), of species that had been introduced into cultivation by the firm before 1808. Andrews (1806: t. 121) was undoubtedly using this binomial for the same plant. However, the binomial had been published eight years earlier by Johann Christoph Wendland (1755–1828) for a different species (Wendland 1798: 45). The persistent use of Andrews’s binomial, despite the priority of Wendland’s, is inexplicable. As noted by Dulfer (1965: 44), Wendland’s name was a synonym of *Ericaconspicua* Sol., which Dulfer relegated to a variety of *E.curviflora* L. (var.splendens (J.C. Wendl.) Dulfer = *E.splendens* J.C. Wendl., non Andrews), but is currently regarded as a distinct species (Oliver and Oliver 2000, 2003; Oliver 2012). There is a later name available to replace *Ericalanata* Andrews and that is *E.flaccida* Hort. ex Link; Sinclair (1825: 10) was the first to make this equation in print. *Ericaflaccida* has been traced in print in several publications (e.g. Anonymous (1808: 191); Cushing (1812: 224; 1814, 224)) and in Conrad Loddiges & Sons’ catalogue for 1811, before it was taken up by Link (1821: 1: 367), who cited English gardeners as his source. None of the sources published prior to 1821 included a diagnosis or description.

#### ﻿ Erica flaccida

Taxon classificationPlantaeEricalesEricaceae

Hort. ex Link, Enum. hort. berol. alt.: 367. 1821

5B74F0FD-5334-570F-851F-916D7607D0FB

Erica lanata Andrews, Heathery, 3: t. 121 (1806); Col. engr. heaths, 3, t. 179. 1809b [wfo-0000672411], nom. illeg., non E.lanata J.C. Wendl., Bot. Beobach.: 45. 1798 [wfo-0000672410]; Salisb., Trans. Linn. Soc. 6: 360 (1802); Guthrie and Bolus, Fl. Capensis 4,1: 73. 1905; Dulfer, Ann. Naturhist. Mus, Wein 68: 112 (1965); Oliver, Strelitzia 29: 499. 2012. Type. Icontype (illustration in Andrews, Heathery, 3: t. 121. 1806 [cit. Dulfer (1965): 112]).

##### Type.

South Africa. “Hab. in Pr. b. sp. [Promontorium Bonae Spei = Cape of Good Hope] … *Hort. angl*.” (B destroyed). Neotype (here designated). South Africa. Western Cape, George Dist., Outeniqua Pass. June 1960. *E.G.H. Oliver 1596* NBG [NBG0112414-0].

### ﻿11. *Ericaniveniana* Hort. ex Loudon (1830), non E.G.H.Oliv. (2000)

This binomial appeared in print two centuries ago (Donn 1804: 69) and was repeated four years later (Anonymous 1808: 193); neither publication contained a diagnosis. However, Loudon (1830: 147) provided a description and explicitly attributed it to Andrews’s “heaths 2” (i.e. “Coloured Engravings of Heaths” 2: t. 112. 1802). The name also occurs in at least two mid-19^th^ century publications: M’Intosh (1855: 709) and Hereman (1868: 219).

Andrews (1802) did not employ the termination –*iana* (adjectival), but published *E.nivenia* (an incorrect variant of the substantive form nivenii) (see Nelson and Oliver (2004: 140)). These epithets are deemed (under ICN (Shenzhen Code) (2018, Art. 61.1 and 61.2); Turland et al. (2018)) to be simple orthographical variants, with the same type, of *E.nivenii*. Regrettably, this means that Oliver’s binomial, proposed when *Syndesmanthusnivenii* N.E. Br. was transferred into *Erica* (Oliver 2000: 225), is an illegitimate later homonym.

The new name alludes to the silky hairs that give the inflorescences a fluffy appearance (Oliver 2012: 486).

#### ﻿ E. bombycina

Taxon classificationPlantaeEricalesEricaceae

E.C.Nelson & Pirie nom. nov.

5A806A8B-4621-566A-9544-5C5E145A846D

urn:lsid:ipni.org:names: 77327525-1

pro E.niveniana E.G.H.Oliv., Contrib. Bolus Herb. 19: 225. 2000 [wfo-0000672716], nom. Illeg., non E.niveniiHort. Ex Loudon (as “nivenia”), Hort. Brit.: 147. 1830 [wfo-1000053516] (see Nelson and Oliver (2004)).

##### Type.

South Africa. Without locality [“Erica N95 on elevated Situations”], *J. Niven 95* (holotype K (K-000225736 [https://powo.science.kew.org/taxon/urn:lsid:ipni.org:names:1017329-1]).

### ﻿12. *Ericaperlata* G.Sinclair (1825), non Benth. (1839)

It appears that no-one has hitherto noticed the inconsistency pertaining to the application of the binomial *Ericaperlata* (meaning “beset with pearls”) (Baker and Oliver 1967: 74). Twentieth-century works consistently attribute the name to Sinclair (1825) and apply it to an erect shrublet, up to 0.6 m tall, with small, urn- to bell-shaped, hairy, white flowers, possessing partly exserted anthers (Baker and Oliver 1967: t. 67; Schumann et al. 1992: 115; Oliver and Oliver 2000, 2003; Oliver 2012: 504). However, Sinclair’s protologue (1825: 18) clearly applies to a different species. He wrote:

**Table:** 

Fol. 4	Bractea. rem.	Anth. sub. ex. mut.	Pistill. ex.	Inflorescentia term. br. umbel.; corol. glob.	Color. Corol. R.P.1-3. Anthers R.R.O.7.	T. Flor. Spring, Autumn.

In other words: leaves ternate; bracts remote; anthers subulate, exserted, muticous; pistil exserted, inflorescences terminal, branching, umbellate; corolla globose, pink-madder; anthers red (between scarlet and Indian red); flowering [in Northern Hemisphere] spring and autumn.

No herbarium specimens from the Duke of Bedford’s Woburn collection, the basis of Sinclair’s descriptions, are known that could be considered as type material, so the protologue is paramount. At least the colour of the flower signals that the protologue does not match the current application of the binomial. Sinclair’s colour codes were very precise, being determined using a specially constructed “diagram of colours” (a colour-wheel) (Sinclair 1825: [39]–41; see Nelson (2011: 8), fig. 5).

Dulfer (1965: 74–75, 77, 141, 153) succeeded in making the various 19^th^-century applications of the binomial more confusing, although he correctly accorded priority to Sinclair, albeit quoting an incorrect publication date (“1816?”) for “Hortus ericaeus Woburnensis” (see Nelson (2003)), an error inadvertently repeated in Baker and Oliver (1967: 74).

According to Dulfer (1965: 74), the disparate species that were identified under this name by various authors included the European *Ericalusitanica* Rudolph (Regel 1843: 162) and his own *E.sphaeroidea* Dulfer (“Lee sec Kl[otzsch] sec Benth. [1839: 672]”) (illustrated by Schumann et al. (1992: 119)). In synonymy under *E.perlata* G.Sinclair, Dulfer (1965: 75) placed E.barbatavar.minor Andrews, *E.pura* Lodd., *E.procumbens* Lodd., *E.ephemera* Tausch, *Gypsocallisprocumbens* G.Don and *Ericodesminus* Kuntze. He concluded (Dulfer 1965: 75): “*E.perlata* ist eigentlich nur eine in allen Teilen kleinere Form von *E.pannosa* und daher kaum eine Art, sondern eine Var. von *E.pannosa*.”

Baker and Oliver (1967: 74–75) were not in any doubt that *Ericaperlata* possessed “pearly-white flowers”, inhabited the Riviersonderend Range and was allied to *E.barbata* Benth., but considering Sinclair’s protologue (1825: 18), his name cannot apply to that plant. In fact, the white-blossomed Riviersonderend heath appears to have no valid name because Sinclair’s is not applicable and renders Bentham’s illegitimate. *Ericapura*Loddiges (1817: t. 72) is a *nomen nudum*, while Loddiges’s *E.procumbens* (1833: t. 1993) is another illegitimate later homonym (contrary to Taylor (2016: 1127) who was not aware of the homonymy).

We propose the replacement name *Ericagalantha* for the Riviersonderend species, in allusion to the white, pearl-like flowers. *Galanthos*, from γάλα (gala = milk) and ἄνθος (anthos = flower), means with a milk-white flower (as in *Galanthus* L., Amaryllidaceae, the Eurasian snowdrop).

#### ﻿ Erica galantha

Taxon classificationPlantaeEricalesEricaceae

E.C.Nelson & E.G.H.Oliv. nom. nov.

FBE672F4-421D-5575-A215-7306EFDBD2C4

urn:lsid:ipni.org:names:77327526-1

pro E.perlata Benth., Prodr. [A. P. de Candolle] 7(2): 670. 1839 [wfo-1000053517], nom. Illeg., non E.perlata G.Sinclair, Hort. Eric. woburn.: 18 (1825) [wfo-0000672873].

##### Type.

South Africa, “In colonia capensi”, *Drège s.n.* (lectotype W, effectively designated by Dulfer (1965: 75)) [https://plants.jstor.org/stable/10.5555/al.ap.specimen.w0008571].

### ﻿13. *Ericapraecox* Hort. Ex G.Sinclair (1825), non Klotzsch (1838)

This binomial was printed, without accompanying descriptions, in Conrad Loddiges & Sons’ catalogue for 1807 and in at least two other pre-1825 publications (Anonymous 1808: 195; Cushing 1814: 210). The next publication, that of Sinclair (1825: 22), is the significant one. As he often did, Sinclair attributed this name to horticulturists, in this case specifically to one, “Hortul. Cormack”, undoubtedly the English nurseryman John Cormack of New Cross, Kent, with whom Sinclair was to enter partnership in 1827 (Harvey 1973, 1981). Messrs Lee & Kennedy had employed the name “Ericapraecox” in the manuscript list of species, mentioned previously (Nelson and Oliver 2004: 138), that the firm had introduced into cultivation.

Sinclair’s (1825: 19) accompanying description is, like the majority of his descriptions, precise and accurate, sufficient to validate the binomial and render Klotzsch’s one illegitimate.

Klotzsch (1838) described a quite different species under this same binomial. His name is currently applied to a spreading, compact shrublet, to 0.2 m tall, bearing urn-shaped to tubular urn-shaped, pink flowers between December and February (Oliver and Oliver 2000, 2003: 443; Oliver 2012: 505). It inhabits mountain summits and ridges between the Du Toitskloof Mountains and Villiersdorp and was illustrated by Schumann et al. (1992: 88, 89).

Dulfer (1965: 60) placed *Ericapraecox* Klotzsch in synonym under E.ventricosavar.meyeriana Dulfer and noted *E.behen* E.Mey. ex Klotzsch as a synonym. However, Klotzsch (1838), noting *E.behen* as a name used in Drège’s Herbarium, gave it as synonymous with *E.savileae* Andrews.

Our replacement epithet refers to the species’ occurrence high in the mountains – above the clouds.

#### ﻿ Erica supranubia

Taxon classificationPlantaeEricalesEricaceae

E.C.Nelson & Pirie nom. nov.

44550228-0B17-52FD-91EF-58BDB61C40D6

urn:lsid:ipni.org:names: 77327527-1

pro E.praecox Klotzsch, Linnaea 12: 517. 1838 [wfo-0000672980], nom. illeg., non Hort. ex G.Sinclair, Hort. eric. woburn.: 19, 32. 1825 [wfo-1200010024]; Benth., Prodr. [A. P. de Candolle] 7(2): 678. 1839.

##### Type.

South Africa. “Dutoitskloof”, *Drège 1147* (lectotype P (P-00110863), designated here (det. E.G.H. Oliver) [https://plants.jstor.org/stable/10.5555/al.ap.specimen.p00110863]; isolectotype W [https://plants.jstor.org/stable/10.5555/al.ap.specimen.w18890186298, https://plants.jstor.org/stable/10.5555/al.ap.specimen.w18890158225].

### ﻿14. *Ericarhodantha*Regel (1842), non Guthrie and Bolus (1905)

Regel (1842, 1843) published this name for a plant which Dulfer (1965: 154) equated with *Ericapelviformis* (= *E.mauritanica*) (see above no. 4). The later publication of the same binomial by Guthrie and Bolus (1905) is illegitimate.

The plant described and named by Guthrie and Bolus (1905) is poorly represented in herbaria, but is listed amongst the currently recognised plant species of southern Africa (Oliver and Oliver 2003: 444). The type locality was Garcia’s Pass and the taxon has recently also been collected on the lower, northern slopes of the Langeberg (Oliver and Oliver 2003: 444). *Ericarhodantha* Guthrie and Bolus resembles *E.gillii* Benth., but can be distinguished from that species by its unridged, brown anthers (ridged and black in *E.gillii*) (Oliver and Oliver 1994: 27). It is an erect shrublet with small, subcalycine, cup-shaped, rose-coloured flowers (Oliver and Oliver 2000).

The new epithet continues the allusion to the rose-red (*rhodellus*) flowers.

#### ﻿ Erica rhodella

Taxon classificationPlantaeEricalesEricaceae

E.C.Nelson & E.G.H.Oliv. nom. nov.

C429FD50-DC89-5601-8FBB-E79176D3175C

urn:lsid:ipni.org:names: 77327528-1

pro E.rhodantha Guthrie and Bolus, Fl. Capensis 4,1: 288. 1905 [wfo-0000673111], nom. illeg., non Regel, Verh. Vereins Beförd. Gartenbaues Königl. Preuss. Staaten 16: 318. 1842 [wfo-1000053518], Regel, Kult. Aufz. Eriken, 158 (1843); Dulfer, Ann. Naturhist. Mus, Wein 68: 154. 1965.

##### Type.

South Africa. Riversdale Div.; Garcias Pass, 1200 ft, *Galpin 3706* (lectotype BOL, effectively designated by Dulfer (1965: 131) [https://plants.jstor.org/stable/10.5555/al.ap.specimen.bol137442]; isolectotypes K [https://plants.jstor.org/stable/10.5555/al.ap.specimen.k000314992], NBG [https://plants.jstor.org/stable/10.5555/al.ap.specimen.nbg0199737-0], PRE [https://plants.jstor.org/stable/10.5555/al.ap.specimen.pre0309255-0], SAM [https://plants.jstor.org/stable/10.5555/al.ap.specimen.sam0010474-0], W [https://plants.jstor.org/stable/10.5555/al.ap.specimen.w19610016719].

### ﻿15. *Ericarugata* Hort. ex G.Sinclair (1825), non E.G.H.Oliv. (2000)

This binomial was printed, without accompanying descriptions, in Conrad Loddiges & Sons’ catalogue for 1811 and in at least two other pre-1825 publications (Cushing 1814: 227; Link 1821: 374). The next publication, that of Sinclair (1825: 22), is, as before, the significant one. Sinclair attributed this name to gardeners (“Hortulanis”), noting it in synonymy under the entry for *Ericarugosa* Andrews; there is a cryptic (cf. ICN (Shenzhen Code) (2018, Art. 38.14); Turland et al. (2018)) reference to Andrews’s publication “Heaths, vol. iv.” (i.e. “Coloured Engravings of Heaths” 4: t. 267 (post 1809; see Cleevely and Oliver (2002))). Andrews’s *E.rugosa* is regarded as a horticultural hybrid.

Unaware of the previous use of this binomial – it is not listed in botanical indexes such as “Index Kewensis”, nor was it noted by Dulfer (1965) – Oliver (2000: 368) chose and published the same binomial when transferring *Coccospermarugosum* Klotzsch into *Erica*. The new epithet alludes to the rugose ovary of this species which is always bi-locular (fide Oliver (2000: 369)).

#### ﻿ Erica didymocarpa

Taxon classificationPlantaeEricalesEricaceae

E.C.Nelson & E.G.H.Oliv. nom. nov.

9773E230-5C18-5C7B-8DC1-EB10980FF393

urn:lsid:ipni.org:names: 77327529-1

pro E.rugata E.G.H.Oliv., Contrib. Bolus Herb. 19: 368 (2000) [wfo-0000673157], nom. illeg., non Hort. ex G.Sinclair, Hort. eric. woburn.: 22 (1825) [wfo-1000053519] (= E.×rugosa Andrews [wfo-0000673159]).

##### Type.

South Africa. “Cap, im Gebirge bei der Kapstadt” [mountains near Cape Town] [loc. 84], *Zeyher s.n.* (lectotype K, designated by Oliver 2000); isolectotype MEL [https://plants.jstor.org/stable/10.5555/al.ap.specimen.mel623226]).

### ﻿16. *Ericaspectabilis* C.F. Waitz (1805), non Klotzsch ex Benth. (1839)

The name *Ericaspectabilis* appeared in print more than thirty years before its publication by Bentham (1839). Under his *E.spectabilis*, Waitz (1805: 220) quoted Andrews’s description making *E.formosa* Andrews a synonym, an equation he reinforced in the “Alphabetisches Verzeichniß der Heidenarten” (Waitz 1805: 324). Andrews’s name was illegitimate, because of the prior publication of *E.formosa* Thunb. and the plant concerned, which possessed vermilion-coloured flowers, is regarded as being a horticultural hybrid (it was claimed by Messrs Rollisson of Tooting).

As the variant “spectabilia”, the binomial has been traced in the 1804 catalogue issued by Conrad Loddiges & Sons who corrected the spelling to “spectabilis” in 1818.

The handsome, variable, white-, cream- to green-flowered species for which Bentham (1839: 659) published the same binomial is restricted to the limestone hills near the coast from Bredasdorp to Gouritsmond, whilst a similar species, *E.syngenesia* Compton, with larger cream-white flowers occurs inland, from the Witteberg to Swartberg (Oliver 2012) and Kammanassie Mountains (Oliver et al., in prep.). Both species were illustrated by Schumann et al. (1992: 156), *E.syngenesia* from the Klein Swartberg (Schumann et al. 1992: 156, figs 12, 13 and 14) and *E.oraria* (as *E.spectabilis*) from coastal habitats near Still Bay (Schumann et al. 1992: 156, figs 10 and 11). *E.syngenesia* and *E.oraria* (as *E.spectabilis*) are included amongst the currently recognised plant species of southern Africa (Oliver and Oliver 2003: 445; Oliver 2012).

The new epithet, from the Latin *ora* (edge or sea coast), reflects the coastal distribution of the species.

#### ﻿ Erica oraria

Taxon classificationPlantaeEricalesEricaceae

E.C.Nelson & E.G.H.Oliv. nom. nov.

72A27749-67A4-513D-9F16-D278D7FDC826

urn:lsid:ipni.org:names: 77330003-1

pro E.spectabilis Klotzsch ex Benth., Prodr. [A. P. de Candolle] 7(2): 659. 1839 [wfo-0000673283], nom. illeg., non Waitz, Beschreibung der Gattung und Arten der Heiden: 220. 1805 [wfo-0000673282]; Guthrie and Bolus, Fl. Capensis 4,1: 57. 1905.

##### Type.

South Africa. “in Strandweld [Strandveld]”, *Drège s.n.* (syntypes: †B, GDC [https://plants.jstor.org/stable/10.5555/al.ap.specimen.g00465165], HBG [https://plants.jstor.org/stable/10.5555/al.ap.specimen.hbg507932], K, W [https://plants.jstor.org/stable/10.5555/al.ap.specimen.w18890186305, https://plants.jstor.org/stable/10.5555/al.ap.specimen.w18890321973, https://plants.jstor.org/stable/10.5555/al.ap.specimen.w0005951]).

### ﻿17. *Ericastenantha*Sweet (1830), non Klotzsch ex Benth. (1839)

Sweet (1830: 340) published this binomial with a reference to the fourth volume of Andrews’s “heath.” and also the synonym “*tenuiflora* γ *carnea*. A. H. v. 4.” (i.e. “Coloured Engravings of Heaths” 4: t. 281 (post 1824)); this indirect reference to a previously published description validates Sweet’s binomial (ICN (Shenzhen Code) (2018, Art. 38.13); Turland et al. (2018)). Thus, Sweet was raising Andrews’s Ericatenuifloravar.carnea to the rank of a species with this binomial.

As noted by Dulfer (1965: 61), following Andrews’s *Ericatenuiflora*, *E.stenantha* Sweet is a synonym of *E.cylindrica* Thunb. Dulfer (1965) did not recognise that Bentham’s binomial (1839: 685) was a later, illegitimate homonym. The species named *E.stenantha* by Bentham (1839) inhabits the upper slopes of the Langeberg; it is an erect shrub, with small, calycine, cup-shaped, dark pink flowers (Oliver and Oliver 2000) and is listed amongst the currently recognised plant species of southern Africa (Oliver and Oliver 2003: 446).

The new epithet is derived from Latin *poculus* (cup) and alludes to the cup-shaped flowers.

#### ﻿ Erica poculiflora

Taxon classificationPlantaeEricalesEricaceae

E.C.Nelson & E.G.H.Oliv. nom. nov.

5BAC75C9-62B4-5F05-89D1-8B77D00EDF33

urn:lsid:ipni.org:names:77327531-1

pro E.stenantha Benth., Prodr. [A. P. de Candolle] 7(2): 685. 1839 [wfo-0000673321], nom. illeg., non Sweet, Hort. Brit.: 340. 1830 [wfo-0000673320] (= E.cylindrica Thunb.).

##### Type.

South Africa. “Berge bei Zwellendam“, *C.F.Ecklon and C.L.P. Zeyher 221* (lectotype W, effectively designated by Dulfer 1965: 130) [https://plants.jstor.org/stable/10.5555/al.ap.specimen.w0005950]; isolectotypes MEL [https://plants.jstor.org/stable/10.5555/al.ap.specimen.mel2384382]; S [https://plants.jstor.org/stable/10.5555/al.ap.specimen.s08-6141]).

### ﻿18. *Ericatenuis*Moench (1802), non Salisb. (1802)

According to Stafleu and Cowan (1981), the supplementary volume to Moench’s “Methodus plantas Horti Botanici et Agri Marburgensis” was issued on 2 May 1802, a little more than three weeks before Salisbury’s paper was published in “Transactions of the Linnean Society” between 24 and 27 May 1802. Thus, Moench’s binomial renders Salisbury’s illegitimate.

Mysteriously, Dulfer (1965: 141) stated that Moench’s name was a synonym of “*Ceramiatenuis* G. Don sec. Benth., Pr. 693 (1838) [sic]”. However, Don’s publication contains no such name, nor is there any reference on p. 693 in Bentham (1839) to this synonymy. The identity of the plant described by Moench, therefore, remains unknown.

**Figure 1.**
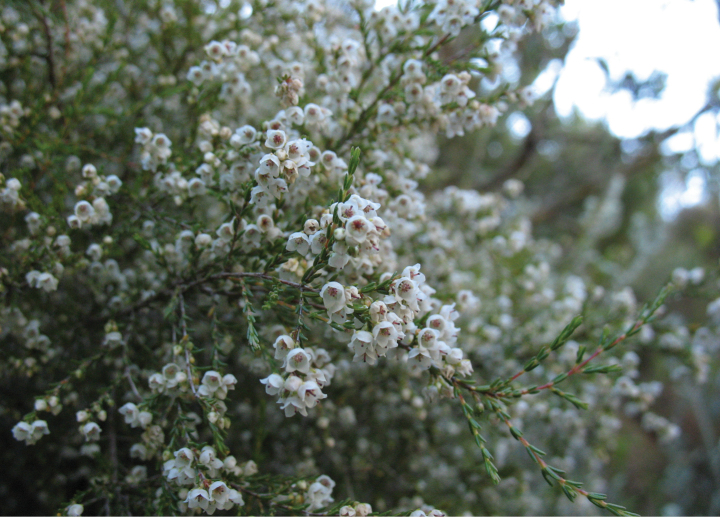
*Ericaoliveranthus* E.C. Nelson and Pirie, renamed in honour of E.G.H. (Ted) Oliver and Inge M. Oliver (photo: MDP; https://www.inaturalist.org/observations/19098927).

*Ericatenuis* Salisb. is the current name for a white-flowered heath (Fig. 1) that occurs in the Western Cape from Clanwilliam to Humansdorp (Schumann et al. 1992: 172), but it must now be replaced.

The new epithet published here is a tribute to our co-author, Dr E.G.H. (‘Ted’) Oliver, pre-eminent authority on the taxonomy of *Erica*, and to his late wife and collaborator, Inge Magdalene Oliver (1947–2003), who was an authority on *Erica* in her own right. They have previously been honoured separately in *E.ingeana* E.G.H. Oliv. and *E.oliveri* H.A.Baker (Schumann et al. 1992: 131).

#### ﻿ Erica oliveranthus

Taxon classificationPlantaeEricalesEricaceae

E.C.Nelson & Pirie nom. nov.

51366B16-7B01-5D74-B044-77985621626D

urn:lsid:ipni.org:names:77327532-1

pro E.tenuis Salisb., Trans. linn. Soc 6: 329. 1802 [wfo-0000673389], nom. illeg., non Moench, Methodus: 17. 1802 [wfo-0000673388].

##### Type.

Without locality or collector, *Ex herb. R. A. Salisbury* (lectotype K [K000314799] [https://powo.science.kew.org/taxon/urn:lsid:ipni.org:names:329729-1]).

### ﻿19. *E.tomentosa*Masson (1776), non Salisb. (1802)

Masson only published one account of his botanical explorations at the Cape of Good Hope (Masson 1776) and, in this, he recorded that on 30 December 1773, during his second journey (Masson 1776: 298–299; see Bradlow (1994: 124)), he reached:

... the hot bath, which is situated at the foot of a ridge of dry mountains: ... Next morning, we went up to the top of this ridge of mountains ... We found here a species of heath remarkable for having its branches and leaves all covered with a fine hoary down or nap, which we thought singular in that genus: we called it *Ericatomentosa*.

Bradlow (1994: 157 n. 212) identified the “hot bath” as the spring situated 4 km east of the southern entrance to Toorwater Poort, in the Groot Swartberg Range. Thunberg was with Masson on this occasion and his corresponding specimen became the type of *Ericapasserina*Montin (1775) (fide J.P. Rourke, in Bradlow (1994: 157 n. 212)).

It is often difficult to decide whether a sentence such as Masson’s constitutes a diagnosis as defined in the International Code of Nomenclature: ‘... a statement of that which, in the opinion of its author, distinguishes the taxon from others’ (ICN (Shenzhen Code) 2018, Art. 38.2; Turland et al. 2018). Given that, at this time, the early 1770s, only about fifty *Erica* species from the Cape Region had been described (Oliver 2000: 4, figure 1; Nelson and Oliver 2004) and that Masson was, by then, familiar with many more undescribed species in their wild habitats, the clause “which we thought singular in that genus” suggests that this is precisely what Masson wrote. Thus, his binomial was validly published. However, it is a junior synonym of *E.passerina*.

It follows that Salisbury’s binomial is illegitimate and has to be replaced. The heterotypic synonym, *Ericavelutina* Bartl. (fide Dulfer (1965: 67)), may be employed for this taxon. It is found on the rocky, lower, southern slopes of the Riviersonderend Mountains (Oliver and Oliver 2000, 2003: 446). An erect shrublet, to 0.5 m tall, *E.velutina* bears small, urn-shaped, finely hairy, lilac or dark pink flowers.

#### ﻿ Erica velutina

Taxon classificationPlantaeEricalesEricaceae

Bartl., Linnaea 7: 645. 1832.

FA12EE73-7DF2-530A-B1B1-4B2A626642BC

Erica tomentosa Salisb., Trans. Linn. Soc. 6: 327. 1802 [wfo-0000673425], nom. illeg., non Masson, Phil. Trans. 66: 299. 1776 [wfo-1000053520] (= E.passerina Montin). Type. South Africa. “Hottentots Holland”, *I. Mulder s.n. ex herb. Salisbury* (not located).

##### Note.

A label identifying the specimen labelled “C.B.S. *Niven 16*” (K-000314197) [https://plants.jstor.org/stable/10.5555/al.ap.specimen.k000314197] as a lectotype is incorrect as the protologue cited only a collection from Hottentots Holland by I. Mulder and the Niven collection is, therefore, not original material.

##### Type.

South Africa. “Am Fusse des Babylonschenthurmbergen [Babilonstoring]”, *Ecklon s.n.* (holotype GOET-003270 [https://plants.jstor.org/stable/10.5555/al.ap.specimen.goet003270]).

## Supplementary Material

XML Treatment for Erica distorta

XML Treatment for Erica umbrosa

XML Treatment for Erica notoporina

XML Treatment for Erica turbiniflora

XML Treatment for Erica concordia

XML Treatment for Erica oresbia

XML Treatment for Erica mallotocalyx

XML Treatment for Erica adelopetala

XML Treatment for Erica flaccida

XML Treatment for E. bombycina

XML Treatment for Erica galantha

XML Treatment for Erica supranubia

XML Treatment for Erica rhodella

XML Treatment for Erica didymocarpa

XML Treatment for Erica oraria

XML Treatment for Erica poculiflora

XML Treatment for Erica oliveranthus

XML Treatment for Erica velutina

## ﻿Additional information

### Author ORCIDs

E. Charles Nelson https://orcid.org/0000-0002-3909-4388

Michael D. Pirie https://orcid.org/0000-0003-0403-4470

### Data availability

All of the data that support the findings of this study are available in the main text.
